# Quantum State Combinatorics

**DOI:** 10.3390/e26090764

**Published:** 2024-09-06

**Authors:** Gregory D. Scholes

**Affiliations:** Department of Chemistry, Princeton University, Princeton, NJ 08544, USA; gscholes@princeton.edu

**Keywords:** quantum states, combinatorics, random graphs, separability, multipartite entanglement

## Abstract

This paper concerns the analysis of large quantum states. It is a notoriously difficult problem to quantify separability of quantum states, and for large quantum states, it is unfeasible. Here we posit that when quantum states are large, we can deduce reasonable expectations for the complex structure of non-classical multipartite correlations with surprisingly little information about the state. We show, with pegagogical examples, how known results from combinatorics can be used to reveal the expected structure of various correlations hidden in the ensemble described by a state.

## 1. Introduction

A point of focus in quantum information science is the states and their properties, because states are the resource that provide quantum correlations like entanglement. By state, we mean the density matrix for a finite-size system relevant to a particular measurement or operation. The state is constructed from a suitable average over the ensemble of systems, states, or measurements, and thus, details about the underlying ensemble are obscured. So, for example, the density matrix of a two-qubit system can be analysed in detail to ascertain whether it is separable or not. Whereas, as the number of qubits increases further, it becomes exponentially harder to decide whether a state is separable or not [1,2,3,4,5].

Here, a different viewpoint is proposed. Rather than trying to quantify entanglement in large and complex systems, it is suggested that properties of a state can be estimated using known results from combinatorics. To start with, recall that a mixed state is produced by some kind of averaging over possible states of the system according to how the state interacts with its environment and how we observe the state. So, a rather bland-looking density matrix (of a large system) hides details of these states within the relevant ensemble. Those states within the ensemble include pure states, but generally, they are states where correlations (like entanglement) can be local compared to the extent of a large system. Thus, there is a more evident structure in correlations within these substates. Now, the approach we will discuss in this paper is based on the notion that instead of analyzing the density matrix to ask global questions about mixedness of the state, we could analyse the underlying structure. We might think that is impossible because the structure is hidden. However, on average, we know what the representative structure almost surely has to be, provided the system is large enough. This is what the theorems of graph theory inform us about.

Specifically, it is proposed that the density matrix of large complex systems can be mapped to a set of random graphs, G(n,p), where *n* is the number of vertices (the dimension of the density matrix), and *p* is the probability that there is an edge connecting each pair of vertices. That is, a specific $p_{ij}$ is the probability of edges connecting vertices *i* and *j*. The vertices of the graph represent the basis states, while the edges indicate correlations between pairs of basis states for a specific state within the ensemble average. We describe how to construct such a map in Section 3. Background information on random graph theory can be found here [6,7]. We can then exploit a rich variety of results known from combinatorics to suggest probable and improbable structures of correlations embedded in the state but hidden by the ensemble average.

Combinatorics concerns counting structures, studying bounding regimes where a property is almost surely found, or finding parameters ensuring certain patterns and properties exist. The methods are particularly impactful for analyzing very large or infinite systems [8]. It is proposed here that we can use this well-developed machinery of combinatorics to analyse the quantum states of large, disordered systems. Why and how would we use these techniques? There are many interesting complex systems that might host quantum correlations, but for such systems, experimental methods for analyzing quantum correlations are out of reach. For instance, quantum-state tomography is incisive, but it is limited to very small systems. It seems more practical to use probabilistic methods to compare expected quantum correlation structure or mixedness in a state. As a first step, here, it is shown that we can infer a surprising amount about a state with very little knowledge about it.

The concept suggesting we need a combinatorial analysis is that there are an extraordinary number of ways that large quantum states can be structured. Instead of enumerating these possibilities, it is more practical to characterize what we expect the correlations to ‘look like’, almost surely. We can then ask how quantum correlations are distributed, whether there might be phases or regimes of interest, or what happens to quantum correlations at various size scales. A key point to recognize is that by elucidating correlation structures encoded by the density matrix, we obtain insight into the make-up of the ensemble underlying a measurement of a complex quantum system. We could also imagine reverse-engineering correlations into the structure of materials.

As stated already, even when given very little information, we can say quite a lot about the structure of correlations likely to be found in a state. As a motivating example, let us ask whether it is likely for a state to be ‘obviously’ separable. All we know is that any possible state on *n* vertices (i.e., *n* basis states) is equally likely. The graphs we discuss throughout the paper enumerate possible correlations. The ensemble of those graphs map to the density matrix, as discussed later in the paper. Thus, disconnected graphs represent states within the ensemble that are ‘obviously’ separable because they represent sets of qubits or basis states that are uncorrelated from each other. (But these are not the only separable states). Is it likely that our state will be ‘obviously’ separable into two or more such distinct sets of qubits?

Assuming unlabelled qubits (that is, we cannot distinguish the vertices), we consider the set of correlation graphs on *n* vertices with no automorphisms. Note that we can estimate the number graphs on unlabelled vertices asymptotically to be (1+o(1))2n2/n!. If n=10, then out of $1.2005\times 10^{7}$ total graphs that show possible correlation maps, only $2.88\times 10^{5}$ are disconnected (see https://oeis.org/A000088 (accessed on 3 June 2024). and https://oeis.org/A001349 (accessed on 3 June 2024)). If n=16, then there are $6.40\times 10^{22}$ total graphs, with merely $3\times 10^{19}$ being disconnected. The proportion of disconnected graphs is small, being about $n2^{1-n}$ for large *n*. This tells us that if we are given a completely random state on a large number of qubits, it is unlikely to be ‘obviously’ separable into distinct sets of qubits.

The structure of entanglement in large systems is complex [9,10,11,12] and underexplored. Here, we investigate how correlation graphs give insight into the expected entanglement structures that are hidden in the state. The probabilistic outlook of this paper might complement theories for the interpretation of quantum mechanics, such as Quantum Bayesianism [13,14,15] (QBism), perhaps even providing a means of clarifying the meaning of a state in the context of the theory. The work might also find applications in event-based reformulations of quantum mechanics [16].

## 2. A Physical Basis for Pairwise Correlations

Although we could proceed with an abstract concept of pairwise correlations, it will help to make the idea concrete. Recall that the graphs represent possible correlations in pure states within the ensemble. Correlations indicated by the graphs can therefore be interpreted in terms of a generalized Schmidt number for multipartite systems. The Schmidt number is a well-known way to quantify entanglement in bipartite pure states [17]. The concept of the Schmidt number has been generalized to multipartite systems in various ways [18,19,20,21]. For example, Guo and Fan [20] have described a hierarchy of Schmidt numbers that quantify the dimensions of entanglement. The strategy involves enumerating the partitions of an *m*-partite system to show how it can be separable in various ways: fully separable, two-separable, three-separable, and so on. This analysis emphasizes that the many states within the ensemble of a mixed state can show a variety of structures. These structures are represented by correlation graphs.

The process of analyzing graphs that represent special pure multiparty quantum states using Schmidt measures has been described in prior work [22]. The present work looks at that problem in reverse—we aim to estimate what graph might be hidden in a general (mixed) state. The generalized Schmidt decomposition relates to these possible pure states within the ensemble. An edge in a graph indicates bipartite entanglement between the vertices (qubits) it connects. In a complete graph on *k* vertices, $K_{k}$ indicates that these *k* vertices (qubits) are genuinely entangled. Any graph can be decomposed into subgraphs of various convex sums of elements, including these kinds of basic structures.

The graphs are not necessarily pure states within the ensemble; rather, they should be thought of as representative correlation maps and are more generally mixed states within the ensemble. For example, a linear graph *u*–*v*–*w* implies that vertex *u* is correlated to *v*, and *v* is correlated to *w*, but *u* is not correlated to *w*. That violates the partial order we would expect for correlations in the state, so it must represent a convex sum of the two pairwise correlations. Thus, the state is separable, even though the graph is connected. Keep in mind that the goal is not to be quantitative, but rather to give a qualitative picture of how the state is structured underneath the ensemble average.

Graph partitions of a four-qubit system can be inferred from Table 3 in ref. [20]. As the number of vertices (qubits) becomes larger, the variety of possible correlation graphs becomes richer. See ref. [23] for drawings of all the connected graphs on six vertices.

**Figure 1:**
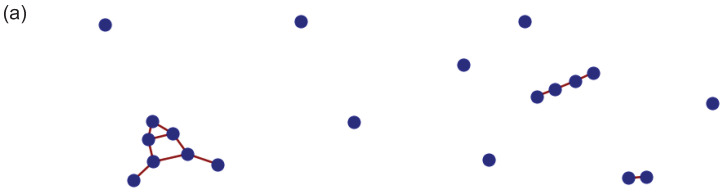

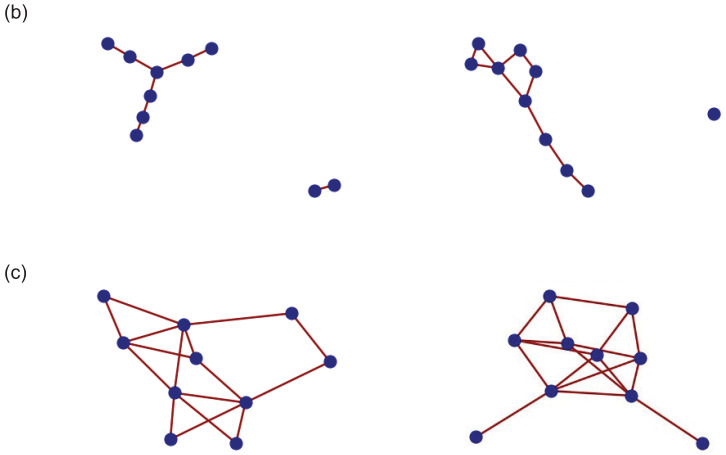
Examples of random graphs G(n,p) on n=10 vertices. (**a**) Here, p=0.1. The disconnected subgraphs show that this state is strongly separable. Note the many isolated vertices. (**b**) Here, p=0.2. The typical graphs tend to have larger connected domains but are mostly strongly separable, like in these examples. (**c**) Here, p=0.3. Most of the graphs are now connected.

In Figure 1, we show some examples of randomly generated correlation graphs representing different strengths of average correlation within the quantum state. Figure 1a shows graphs where p=0.1. The disconnected subgraphs indicate that the ensemble state is strongly separable. Note the many isolated vertices, suggesting very weak correlation. In Figure 1b, the graphs are calculated with p=0.2. The typical graphs tend to have larger connected domains but are mostly strongly separable, like in these examples. In Figure 1c, p=0.3. Most of the graphs are now connected, which is expected based on what we know about the ‘phase transition’ in random graphs [6,24]. Note the diversity of structures, highlighting how the correlations underlying the ensemble are much richer than might be expected by simply inspecting the density matrix. That is, these correlation structures are well disguised by averaging.

## 3. Maps to Graphs Derived from the Density Matrix

We have shown how we can conclude a surprising amount about a state by knowing nothing about it except its size. Methods from combinatorics can be even more powerful if we consider more information from the state’s density matrix, or even just the expected nature of the density matrix (e.g., is the state strongly mixed?). To do that, we need to define an appropriate approximate map, or bounding maps, from the the density matrix on *n* basis states, $\rho_{n}$, to the set of random graphs G(n,p). A prior work studied quantum ensembles of a state defined by the density matrix [25]. Here, we aim to obtain a probabilistic map. We cannot define a precise map because the heterogeneity of states subsumed by the average in $\rho_{n}$ demands a corresponding (convex sum of) heterogeneity of maps. All we really need is a reasonable effective map. Or we can choose maps that bound the likely properties from above and below. One property we do require of the map is that it is size-consistent.

We need to estimate *p* from the entries in $\rho_{n}$. We can assign a distinct $p_{ij}$ mapping from each $\rho_{ij}$ off-diagonal entry, which is a straightforward extension of what we will do here. Here, we will estimate a representative single *p*. This makes the application of theorems easier and is also likely to be the most sensible approach because we only want to estimate expected properties. Indeed, it seems likely that it is sufficient to know how to map very weak, weak, strong, or very strong correlations to an effective *p*. As a starting point for any map, $\rho_{n}$ should be non-negative, so from this point on, we consider only $|\rho_{n}|$. Before proposing a useful map, let us establish bounds.

A simple lower bound is simply the map from off-diagonal entries in $|\rho_{n}|$ to the corresponding $p_{ij}$ in G(n,p). Thus, we might take *p* to be the average of the $p_{ij}$. This is a lower bound for *p* because, for example, the coherent state on *n* vertices with all off-diagonal entries, $\frac{1}{n}$, is a pure state and should correspond to edge probability p=1, so as to generate the complete correlation graph $K_{n}$. Instead, it maps to a set of graphs that almost certainly will not contain $K_{n}$. Note that this map is not size-consistent. A simple, and probably too trivial, upper bound can obtained by normalizing the off-diagonal entries in $|\rho_{n}|$ so that the maximum entry is unity. Then, we can use these values for $p_{ij}$ or their average for *p*.

For a map to be size-consistent, the off-diagonal entries of $|\rho_{n}|$ should be normalized by *n* or a factor that scales appropriately with *n*. A map that uses this concept can be applied to normalize $|\rho_{n}|$ by dividing all entries by the maximum diagonal entry in $|\rho_{n}|$. Then, *p* is estimated as the average renormalized off-diagonal entry of $|\rho_{n}|$. This is the map we will study for the remainder of this section.

First, we test this map on a very small system, the density matrix for certain mixed states in the system of two qubits, labelled A and B. We index the rows and columns of $\rho_{4}$ as ${|0\rangle }_{A}{|0\rangle }_{B}$, ${|0\rangle }_{A}{|1\rangle }_{B}$, ${|1\rangle }_{A}{|0\rangle }_{B}$, ${|1\rangle }_{A}{|1\rangle }_{B}$. Peres [1] wrote the density matrix so that a singlet state makes up a fraction *x* of the mixed state, while the remaining (1−x) fraction is a ‘random fraction’, comprising equal admixture of the singlet and three triplet states to produce a fully mixed fraction of the state:

(1) $$\rho_{\mathrm{mixed}}=\left[\begin{array}{cccc} \frac{\left(1-x\right)}{4} & 0 & 0 & 0\\ 0 & \frac{\left(1+x\right)}{4} & -\frac{x}{2} & 0\\ 0 & -\frac{x}{2} & \frac{\left(1+x\right)}{4} & 0\\ 0 & 0 & 0 & \frac{\left(1-x\right)}{4} \end{array}\right].$$

We know that if x=1, then the state is a pure (singlet) state. Setting x=1 and mapping $\rho_{4}$ to G(n,p) gives an adjacency matrix with entries of 1 or 0, and G(n,p) encodes solely the complete graph $K_{2}$, which is in a pure state, as required. If $x<\frac{1}{3}$, then the state is separable (see ref. [1], or the tutorial explanation in [26]). Let us put $x=\frac{1}{3}$ into $\rho_{4}$; then, the map sets the non-zero edge probabilities to $\frac{1}{2}$, which is a reasonable value for the threshold value. Keep in mind this example is simply a calibration; the methods proposed here are best suited for studying large quantum systems.

To estimate appropriate values of *p*, we performed numerical studies. In a prior work [27], we investigated the states and their mixedness, encoded by *k*-regular random graphs. In particular, we introduced structural disorder by randomly removing some fraction of the edges. The mixedness of the states, as a function of the number of edges deleted, was reported. The emergent state is a pure state, but when sufficient edges are deleted, it merges into the random states. Thus, we have a way of tuning the mixedness of the state. See Figure 2 of ref. [27]. We use the same technique here to produce a series of density matrices with a range of mixedness, which we quantify using the relative entropy of coherence [28]. Quantum relative entropy, $S_{\rho ∥\sigma }$, quantifies the distance of a state ρ from the nearest incoherent (mixed) state σ:

$$S_{\rho ||\sigma }=\mathrm{Tr}\left(\rho \log_{2}\rho \right)-\mathrm{Tr}\left(\rho \log_{2}\sigma \right).$$

The state σ is constructed by setting the off-diagonal entries from the density matrix of ρ to zero. The relative entropy therefore explicitly measures how far a state is from a comparable mixed state.

We have n=600 vertices and k=20; then, we delete a fraction of the total kn/2 edges randomly. For each density matrix, we estimate *p*, the mean pairwise edge probability, as described above: we take the mean of the off-diagonal entries of $|\rho_{n}|$, normalized by the maximum diagonal entry. The mean pairwise edge probability translates to the ratio of actual edges to total possible edges, averaged over all the graphs in the ensemble. For each graph, with the actual number of edges being *m*, the pairwise edge probability is 2m/kn. Notice this is not for edges of any pair of vertices, but only for the pairs that ensure the graph is precisely *k*-regular.

**Figure 2:**
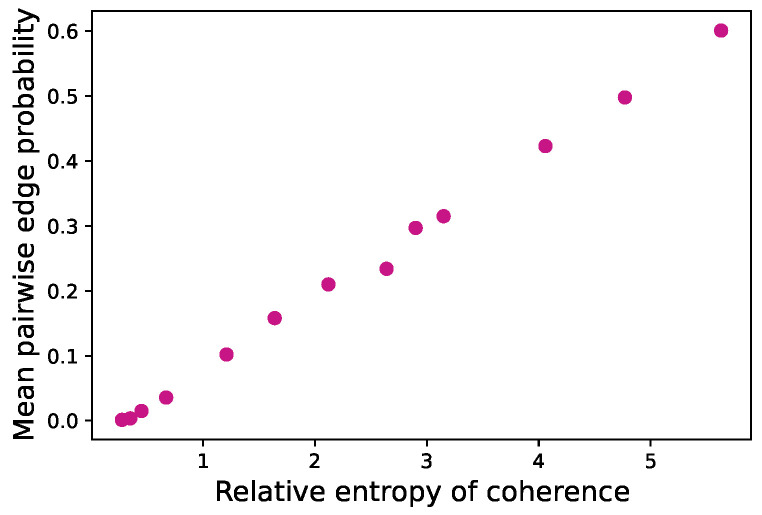
Correlation of the average pairwise probability of correlation *p* with the relative entropy of coherence. Calculations are for an ensemble of *k*-regular random graphs with edge disorder (see text), with the state associated with the largest eigenvalue in the spectrum.

The results are plotted in Figure 2. Notice the almost linear relationship between relative entropy of coherence and the estimated values of *p* for this system. The plot allows us to qualitatively label the regimes of correlations as follows: very weak, p<0.001; weak, p<0.01; medium, 0.01≤p≤0.1; and strong p>0.1 correlations.

## 4. Combinatorial Analysis of States

We start by returning to the question about the likelihood of finding cliques in quantum states—that is, complete correlations among *r* qubits, which are represented by complete subgraphs $K_{r}$. We can use a result stated in Bollobás and Erdös [29]. A clique in a graph is an induced subgraph on *r* vertices that is a complete graph, $K_{r}$. Define $Y_{r}$ to be the number of *r*-cliques in G(n,p). The expectation of $Y_{r}$ is as follows:

(2) E(Yr)=nrpr2.

**Figure 3:**
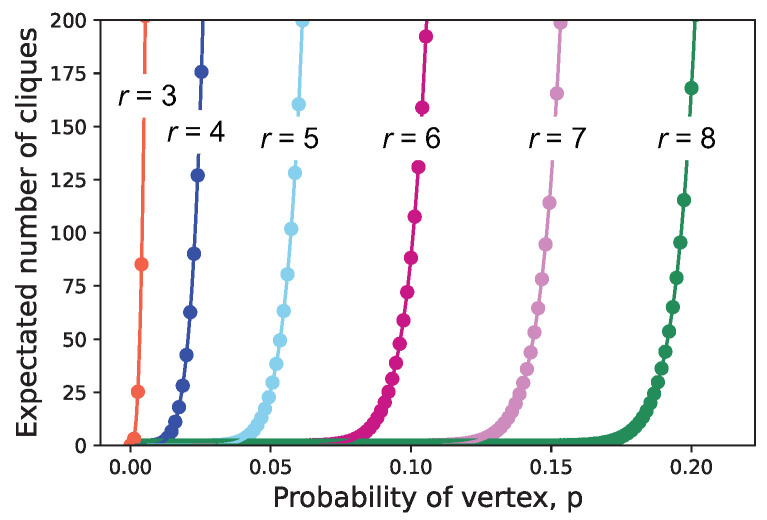
Estimated expected number of cliques as a function of *p* for random graphs G(n,p) on 2000 vertices.

In Figure 3, we plot the expectation of $Y_{r}$ as a function of *p* for a graph with 2000 vertices. The plot highlights the thresholds for onsets of cliques with various sizes. Thus, a distribution of cliques is expected, and its make-up depends strongly on *p*. Clearly, small cliques overwhelm large cliques, meaning that most multipartite correlations will be found among small numbers of vertices.

We may obtain further insight using Ramsey theory [30,31]. Here, we use edge colouring rather than vertex colouring.

**Theorem 1.**  *Two-colour Ramsey’s Theorem for Graphs: Let r,s be any two positive integers. There exists a least positive integer R(r,s), for which every edge colouring of the complete graph on R(r,s) vertices using two colours, say red and blue, contains a blue clique on r vertices or a red clique on s vertices.*

The R(r,s) are known as Ramsey numbers. The classic example is for R(3,3)=6, which can be explained as follows. Take any group of at least six guests randomly at a party. It is certain that either three guests all know each other or that three guests have never met. What Ramsey theory tells us about multipartite correlations is that there is a threshold value (not necessarily sharp) of global correlation such that below the threshold a particular multipartite correlation (a complete correlation graph $K_{r}$) does not exist, but above the threshold, it certainly does exist. For example, let us say we have a quantum state comprising about 100–160 qubits. Given that the Ramsey number R(6,6) lies in the range [102,165], then we know for sure that the state either contains six qubits that are not mutually correlated at all or six qubits that are six-partite entangled. The known values for R(r,s) indicate that large multipartite correlations are not very likely in large states.

For another example we use the following [32]:

**Theorem 2.**  *Erdös and Szekeres: If G is a graph on n vertices, then G contains either a clique or an independent set of size $\geq \frac{1}{2}\log_{2}n$.*

That is, for any system of *n* qubits, at least $\frac{1}{2}\log_{2}n$ qubits are completely correlated among themselves or completely uncorrelated. This example also shows how applications of Ramsey theory can be useful [30].

As an example, if n=100, then the graph contains either a clique or a stable set of size at least 3. How likely is it that the graph contains a stable set of size 3 and no clique of size 3? We can estimate this in the case of labelled vertices using a Corollary found in ref. [33] (stated here as a theorem):

**Theorem 3.**  *Erdös, Kleitman, and Rothschild: Let $G_{k}\left(n\right)$ be the number of graphs with n vertices and with no subgraph of type $K_{k}$. Then,*

$$\log_{2}\left(G_{k}\left(n\right)\right)=\frac{n^{2}}{2}\left(1-\frac{1}{k-1}\right)+o\left(n^{2}\right).$$

Putting k=3 for cliques of size 3 (triangles, $K_{3}$) we can see that the number of triangle-free graphs is asymptotically extremely small compared to the total number of labelled graphs on *n* vertices, 2n2. The same conclusion also holds for unlabelled graphs.

The chromatic number is a property of graphs that tells us something about the global connectivity [34], albeit in a way which means that interpretation depends, to some extent, on the kinds of graphs being studied. Chromatic number and graph colouring give information on sets of vertices that are not directly coupled to each other. Thus, a simple, general idea is that the larger the chromatic number, the more connected the graph (the more correlations). In fact, this is not strictly true [35], but it can be a good guide if used carefully. We start with some definitions [36].

**Definition 1.**  *Vertex colouring: Let G be a graph on V vertices labelled v,w,⋯. A vertex colouring of G is a map c:V→S such that c(v)≠c(w) whenever v and w are adjacent. The elements of the set S are the colours.*

**Definition 2.**  *Chromatic number: We find the smallest integer k such that G has a k-colouring. That is, the set S has a minimum size of |S|=k. Then, k is the chromatic number of G, written as χ(G).*

**Figure 4:**
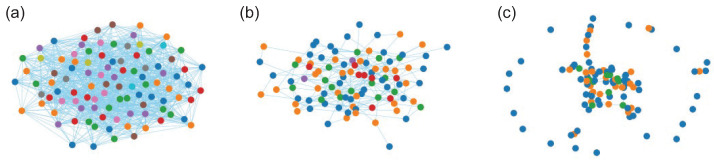
Randomly generated graphs on 100 vertices, G(n,p), with vertices coloured using a greedy colouring algorithm. (**a**) p=0.2; chromatic number χ(G)=10. (**b**) p=0.05; chromatic number χ(G)=5. (**c**) p=0.02; chromatic number χ(G)=3.

The chromatic number of random graphs has been studied, and its expectation can be bounded [37,38]. Alon and Krivelevich [39] have shown how various regimes of random graphs have remarkably similar chromatic number values. In fact, it is easy to see with numerical experiments that for any given *n* and *p*, there is not a wide variation in χ(G(n,p)). In Figure 4, we plot three graphs randomly selected for n=100, and values of *p* are indicated. The vertex colouring is shown in the plots, and the chromatic number of each graph is noted. These graphs are typical of others randomly generated with these parameters, as can be easily checked.

Graphs can have remarkably intricate structures, which are often the basis for conjectures and theorems. Quantum states, therefore, contain related subtle substructures. For instance, see the following [40]:

**Theorem 4.**  *Kühn and Osthus: For every k there exists d=d(k). This means that every graph G with an average degree of at least d contains a subgraph of average degree of at least k whose girth is at least six.*

That is, we will find a subgraph that contains the shortest cycle of ≥6, provided that *d* for the graph *G* is sufficiently large. The result is surprising because, intuitively, we expect that subgraphs with small girth will dominate when the average degree is large, as suggested by the results shown in Figure 3.

The following conjecture has led to many interesting examples of induced subgraphs that are certain to be found in graphs with a large chromatic number [41,42,43]. Let *H* be any graph, and let G(H) denote the set of all graphs not containing *H* as an induced subgraph. If *F* is a forest, does there exist a function $f_{F}$ such that

(3) $$\chi \left(G\right)\leq f_{F}\left(\omega \left(G\right)\right)$$

for all G∈G(F)? ω(G) denotes the size of a maximum complete subgraph of *G* (the clique number).

A graph *H*-consistent with the conjecture is termed an χ-bounding graph. So, here, the assertion is that every forest is χ-bounding, which is true only when all the components of the forest—the trees—are χ-bounding.

Examining this conjecture [44,45] has led to the identification of special kinds of trees for which the conjecture holds—aptly named caterpillars, brooms, stars, and so on. These subgraphs must conversely be found in graphs that have a large chromatic number [46], specifically graphs where

(4) $$\chi \left(G\right)>f_{F}\left(\omega \left(G\right)\right).$$

Recognizing that structure can be built into ‘random’ graphs, it may be interesting to inquire whether we can invert our analysis. That is, can we design disordered quantum materials that encode certain non-trivial correlation structures? How would that influence the properties of those materials?

## 5. Conclusions

There are an extraordinary number of ways that large quantum states can be structured. Here, we made the case that, instead of enumerating these possibilities explicitly, it can be more practical to characterize what we expect the correlations to ‘look like’. We can then ask how quantum correlations are distributed, whether there might be phases or regimes of interest, or what happens to quantum correlations at various size scales. A key point to recognize is that by elucidating correlation structures encoded by the density matrix, we obtain insight into the make-up of the ensemble (of not necessarily pure states) underlying a measurement of a complex quantum system. We could also integrate reverse-engineering correlations into the structure of materials. We showed in this paper that by using the results from combinatorics, certain properties of the expected structure of correlations can be revealed with remarkably little information about the state. Finally, it may come as a surprise to realize there is so many interesting structural features in ‘random’ graphs and, therefore, large quantum states, even when correlations are weak. However, there is order hidden in almost all random systems if they are sufficiently large. For example, arithmetic progressions have been widely studied. One recent paper [47], for example, improved the bound on the size of a set of integers, guaranteeing that it contains a three-progression (i.e., *a*, a+b, a+2b). Finally, the approach described in this paper might inspire ways of estimating the properties and structure of correlations in very large and complex systems, where quantitative experimental methods like state tomography are impractical.

## Data Availability

The original contributions presented in the study are included in the article, further inquiries can be directed to the corresponding author.
